# In-Depth Characterization of Protein Disulfide Bonds by Online Liquid Chromatography-Electrochemistry-Mass Spectrometry

**DOI:** 10.1007/s13361-015-1258-z

**Published:** 2015-09-14

**Authors:** Linda Switzar, Simone Nicolardi, Julie W. Rutten, Saskia A. J. Lesnik Oberstein, Annemieke Aartsma-Rus, Yuri E. M. van der Burgt

**Affiliations:** Department of Human Genetics, Leiden University Medical Center (LUMC), Leiden, The Netherlands; Center for Proteomics and Metabolomics, Leiden University Medical Center (LUMC), Leiden, The Netherlands; Department of Clinical Genetics, Leiden University Medical Center (LUMC), Leiden, The Netherlands; Albinusdreef 2, Postzone S3, P.O. Box 9600, 2300 RC Leiden, The Netherlands

**Keywords:** Protein disulfide bonds, Electrochemistry, High resolution mass spectrometry

## Abstract

Disulfide bonds are an important class of protein post-translational modifications, yet this structurally crucial modification type is commonly overlooked in mass spectrometry (MS)-based proteomics approaches. Recently, the benefits of online electrochemistry-assisted reduction of protein S–S bonds prior to MS analysis were exemplified by successful characterization of disulfide bonds in peptides and small proteins. In the current study, we have combined liquid chromatography (LC) with electrochemistry (EC) and mass analysis by Fourier transform ion cyclotron resonance (FTICR) MS in an online LC-EC-MS platform to characterize protein disulfide bonds in a bottom-up proteomics workflow. A key advantage of a LC-based strategy is the use of the retention time in identifying both intra- and interpeptide disulfide bonds. This is demonstrated by performing two sequential analyses of a certain protein digest, once without and once with electrochemical reduction. In this way, the “parent” disulfide-linked peptide detected in the first run has a retention time-based correlation with the EC-reduced peptides detected in the second run, thus simplifying disulfide bond mapping. Using this platform, both inter- and intra-disulfide-linked peptides were characterized in two different proteins, ß-lactoglobulin and ribonuclease B. In order to prevent disulfide reshuffling during the digestion process, proteins were digested at a relatively low pH, using (a combination of) the high specificity proteases trypsin and Glu-C. With this approach, disulfide bonds in ß-lactoglobulin and ribonuclease B were comprehensively identified and localized, showing that online LC-EC-MS is a useful tool for the characterization of protein disulfide bonds.

**Electronic supplementary material:**

The online version of this article (doi:10.1007/s13361-015-1258-z) contains supplementary material, which is available to authorized users.

**Graphical Abstract:**
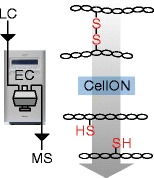
ᅟ

## Introduction

Disulfide bonds are crucial for protein structure and function [1, 2]. As such, changes in the disulfide arrangement have been associated with altered activity of proteins, such as in antibodies [3, 4] and hormones [5, 6]. In the case of protein-based therapeutics, confirmation of a correct formation of disulfide bonds is critical [7]. However, in standard mass spectrometry (MS)-based protein analysis, S–S bonds are cleaved in a chemical reduction step, followed by alkylation of cysteines to achieve more efficient proteolytic digestion and prevent reformation of the disulfide bonds. This results in a loss of information on the location and connectivity of disulfide bonds. Therefore, tailored approaches are required to obtain disulfide bond information and, to this end, various methods have been reported. Early studies, as reviewed in 2002 by Gorman et al. [8], were based mainly on matrix-assisted laser desorption ionization-mass spectrometry (MALDI-MS) in combination with a stable isotope labeling strategy for the analysis of disulfide bonds in either intact or digested proteins. More recent publications employed liquid chromatography (LC)-MS-based methods for bottom-up proteomics [9] and high-resolution MS instruments for top-down analysis [10, 11]. The latter approach requires minimal sample preparation and handling, which is advantageous for disulfide mapping as the risk for so-called reshuffling is reduced. Unfortunately, the presence of disulfide bridges often hampers tandem-MS backbone cleavage of a protein, resulting in a limited protein sequence coverage. Thus, in order to achieve an improved sequence coverage in the top-down analysis of proteins, a chemical reduction of disulfide bonds is often required [12]. Electrochemistry (EC) has been successfully applied as an alternative method for S–S reduction, as the presence of reducing reagents may interfere with chromatographic and MS performance [13]. Not only does EC provide a “clean” approach for disulfide reduction, the electrochemical cell can also be hyphenated with MS for online reduction of disulfide bonds and, moreover, is compatible with different ionization techniques, such as electrospray ionization (ESI)-MS [13] and desorption electrospray ionization (DESI)-MS [14–16]. It was observed that gradual adjustment of the settings of the electrochemical cell leads to partial reduction, which can be applied to the characterization of disulfide bonds [17, 18]. Using this feature and through direct coupling of EC with high resolution ESI-MS, we previously characterized disulfide bonds in oxytocin and hepcidin [19]. This approach can also be applied to larger disulfide bond-containing proteins, such as the structural analysis of a monoclonal antibody [20]. However, it has to be noted that with increased complexity of the protein in terms of molecular weight and number of disulfide bonds, the interpretation and assignment of data resulting from top-down experiments can be difficult or even become ambiguous, stressing the importance of high resolution MS analysis.

Currently, a growing interest is noted with regard to bottom-up approaches for disulfide bond mapping in which proteolysis is performed on non-reduced proteins to keep the disulfide bonds intact. Importantly, disulfide bond reshuffling has been observed following digestion at pH 8 [8, 21], which is the optimal pH for trypsin digestion [22]. Therefore, alternative digestion strategies have been investigated. In one study, it was found that disulfide reshuffling in a mixture of six proteins could be minimized by performing trypsin digestion at a lower pH of 6.8 and was completely absent when using pepsin at pH 1.5. Therefore, pepsin was favored over trypsin [23]. In another study, different (combinations of) specific and nonspecific proteases were examined for protein disulfide mapping, and digestions were performed at pH 6.5 in the presence of *N*-ethylmaleimide to successfully avoid reshuffling [24]. Here, the nonspecific proteases led to the most disulfide bond identifications in low complexity samples ( ≤ 10 proteins), but these were outperformed by combinations of specific proteases for complex protein samples. Pepsin was also favored in a study that combined (online) digestion with EC/DESI-MS for online electrochemical reduction and detection of disulfide-linked peptides [15, 18]. So far, this approach has been applied to relatively simple peptides and small proteins, but it shows potential for the analysis of more complex protein samples. Evidently, proteolytic digestion of a more complex protein (mixture) generates a larger number of peptides, which may lead to several issues with this type of analysis, such as ionization suppression effects and peak overlap in MS and MS/MS spectra. In addition, the use of a nonspecific protease, such as pepsin, yields many short-chained peptides with limited protein sequence information. Moreover, MS/MS spectra of such small peptides ( < 5 amino acid residues) are often difficult to assign unambiguously. Therefore, in the current study, we aimed for protein digestion with specific proteases at a relatively low pH followed by up-front separation of disulfide-containing peptides, in combination with our previous application of EC for the characterization of S–S bridges in proteins. The use of EC also enables the analysis of complex arrangements of disulfide bonds, for example for cysteine-rich proteins, such as the neurogenic locus notch homolog protein 3 (NOTCH3). Protein digestion increases the complexity of the sample, which can be overcome by upfront LC separation, as has previously been performed with (UP)LC/EC/DESI/MS for mixtures of small peptides and proteins [25, 26]. In the current work, a LC-system was coupled to an EC-ESI-Fourier transform ion cyclotron resonance (FTICR)-MS platform to allow online separation, S–S reduction, and mass analysis of disulfide-linked peptides. With this set-up, the link between the disulfide-linked peptide and the reduced, hereafter named “disconnected” peptides was preserved in the retention time dimension, thereby facilitating the characterization of disulfide bonds. This online LC-EC-MS approach was evaluated for disulfide bond analysis in two standard proteins, ß-lactoglobulin and ribonuclease B.

## Experimental

### Chemicals

Ribonuclease B from bovine pancreas, ß-lactoglobulin from bovine milk, ammonium acetate, acetic acid, and formic acid were purchased from Sigma-Aldrich (St. Louis, MO, USA). Sequencing-grade modified trypsin and Glu-C were obtained from Promega (Leiden, The Netherlands). HPLC Supra gradient-grade acetonitrile was purchased from Biosolve (Valkenswaard, The Netherlands). Ultrapure water was supplied from a Elga Purelab ultra system (Ede, The Netherlands).

### Protein Digestion

Trypsin digestion was performed by mixing 10 μL protein solution (10 μg/μL in MilliQ) with 15 μL of a 50 mM ammonium acetate buffer (pH 5.5), 4 μL acetonitrile, and 2 μL trypsin (1 μg/μL), followed by incubation of the mixture overnight at 37°C. The following morning, the sample was supplemented with another 2 μL of trypsin and incubated again for 3 h at 37°C. Finally, 2 μL of formic acid was added to stop the digestion and diluted 3-fold for analysis.

For ribonuclease B, also a double digestion was performed where in the second 3-hour digestion step, trypsin was replaced with Glu-C and the digestion was performed in the presence of a higher percentage of acetonitrile. For this, 10 μL protein solution (10 μg/μL in MilliQ) was mixed with 10 μL of a 50 mM ammonium acetate buffer (pH 5.5), 5 μL acetonitrile, and 2 μL trypsin (1 μg/μL), followed by incubation of the mixture overnight at 37°C. The following morning, 2 μL of Glu-C (1 μg/μL) was added and the sample was incubated again for 3 h at 37°C. After addition of 2 μL formic acid, the sample (35 μL total volume) was evaporated to approximately 20 μL final volume. The samples were stored at – 80°C until analysis.

### EC Optimization for Online LC-MS

Intact ß-lactoglobulin was dissolved (0.2 mg/mL) in 10%, 20%, 30%, or 40% acetonitrile containing 0.25 or 0.5% formic acid and analyzed in flow injection analysis (FIA) mode with an isocratic gradient of the same composition. The electrochemical cell was operated in pulse mode at 45°C using default EC conditions (E1 = – 2.0 V, E2 = 2.0 V, t1 = 2000 ms, t2 = 1000 ms, and ts = 20 ms). The EC parameters E1 (– 0.4 – – 2.6 V), E2 (0.4 – 2.6 V), t1 (1000 – 2000 ms), t2 (500 – 1500 ms), and ts (20 – 160 ms) were then optimized sequentially.

### LC-EC-FTICR-MS Analysis of Protein Digests

An Ultimate3000 LC system (Thermo Scientific, Breda, The Netherlands) was hyphenated to an electrochemical cell with a titanium-based working electrode (μPrep cell, Antec, Zoeterwoude, The Netherlands), which was coupled to the ESI source of a 15 T SolariX XR FTICR MS system equipped with a dynamically harmonized ParaCell (Bruker Daltonics, Bremen, Germany). The equivalent of 2 μg of a tryptic digest was injected onto a Luna RPC18 column (150 × 1 mm, 3 μm, Phenomenex, Utrecht, The Netherlands) and the peptides were separated using a linear gradient starting from 0% B to 40% B in 30 min, then 100% B for 4 min and re-equilibration at 0% B for 11 min. All LC-runs were performed with a flow rate of 50 μL/min to allow for splitless coupling to the μPrep electrochemical cell [17]. The mobile phases consisted of 0.5% formic acid in 5% acetonitrile for A and 0.5% formic acid in 95% acetonitrile for B. The analytical column and the electrochemical cell were kept at 45°C. The first analysis of the digests was performed with the cell switched off (CellOFF mode) to keep the disulfide bonds intact and detect the disulfide-linked peptides. In the second analysis, electrochemical reduction of disulfide bonds was performed using the optimized EC conditions (E1 = – 1.4 V, E2 = 0.4 V, t1 = 2000 ms, t2 = 1000 ms, and ts = 40 ms) resulting in the detection of the disconnected peptides. The flow from the electrochemical cell was directed to the MS from 5 to 34 min and the 26 min MS analysis started at 7 min.

The proteolytic peptides from ribonuclease B and ß-lactoglobulin were analyzed by ESI-FTICR MS in chromatography mode with auto MS/MS. To this end, after each MS scan, three precursor ions were consecutively isolated through the quadrupole (Q1) with an isolation window of 5.0 Da, accumulated in the hexapole collision cell for 3 s and fragmented by collision-induced dissociation (CID). In both MS and MS/MS experiments, ions were measured in the ICR cell from *m/z* 306.80 to 3000.00 using the broadband detection mode with 1 M data points. Precursor ions were excluded after one MS/MS spectrum and released after 0.5 min. Singly charged ions were not selected for CID experiments. The trypsin digest of ribonuclease B was also analyzed by targeted ESI-CID-FTICR MS with the Q1 set to *m/z* 856.5, an isolation window of 8.0 Da, and collision energy of – 25 V. All spectra were visualized and processed using DataAnalysis 4.2 (Bruker Daltonics).

### Data Analysis

Database searches were performed with Mascot (ver. 2.5, www.matrixscience.com) on mzXML files created using CompassXport (ver. 3.0.7, Bruker Daltonics, Bremen, Germany). MS/MS ion searches were performed using trypsin (K,R′) or trypsin/Glu-C (D,E,K,R′) as enzyme with a maximum of two missed cleavages, the SwissProt database (weekly updated), a peptide and MS/MS tolerance of 0.1 Da, ESI-FTICR instrument fragmentation settings, and oxidation (C) and (M) as variable modifications. To check for nonspecific cleavage products, MS/MS ion searches were performed by changing the enzyme to none. Manual de novo sequencing was performed for annotation of MS/MS spectra of disulfide-linked peptides.

## Results and Discussion

### EC Optimization for LC-EC-MS

**Figure 1:**
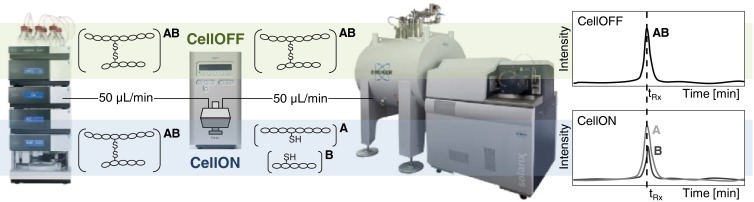
Scheme of the LC-EC-MS platform. Detection of a disulfide-linked peptide (AB) is achieved in CellOFF mode. Disconnected peptides (A and B) are detected following electrochemical reduction in CellON mode

The setup of the LC-EC-MS platform is depicted in Figure 1. The use of an LC-gradient in combination with the electrochemical cell may have an effect on the reduction efficiency because of the continuously changing acetonitrile composition of the mobile phase. Therefore, first a series of FIA experiments were performed to optimize the EC parameters for post-hyphenation of the electrochemical cell to a LC separation. This was done by following the reduction of intact ß-lactoglobulin in different mobile phase compositions and varying EC conditions. In these experiments, it was observed that an increased percentage of acetonitrile ( ≥ 30%) resulted in a higher charge state, which is commonly rationalized by increased unfolding of the protein at non-native spray conditions [27]. However, upon activation of the electrochemical cell, the observed charge state distributions were rather similar in all evaluated percentages of acetonitrile (see Supplemental Material, Figure S1a). Electrochemical reduction of the disulfide bridges may result in protein unfolding, thus explaining a higher charge state independent of the organic solvent content. The reduction itself is further confirmed by a mass shift in the isotope pattern, which was found to be independent of the percentage of acetonitrile (see Supplemental Material, Figure S1c and d). Furthermore, changing the percentage of formic acid in the mobile phase did not affect disulfide bond reduction since similar mass shifts were obtained for both evaluated formic acid percentages under reducing conditions of the electrochemical cell. It was noted that the percentage of formic acid in the mobile phase did have an effect on protein charge state distribution, in the sense that a higher charge state was obtained with a higher percentage of formic acid. The EC conditions were thus optimized at a percentage 0.5% formic acid for each of the different percentages of acetonitrile in the mobile phase. Only minor differences in optima for maximum charge state and reduction were observed and the following settings worked well for all mobile phase compositions: E1 = – 1.4 V, E2 = 0.4 V, t1 = 2000 ms, t2 = 1000 ms, and ts = 40 ms. (see Supplemental Material, Figure S1b).

### LC-EC-FTICR-MS Analysis of Trypsin Protein Digests

**Figure 2:**
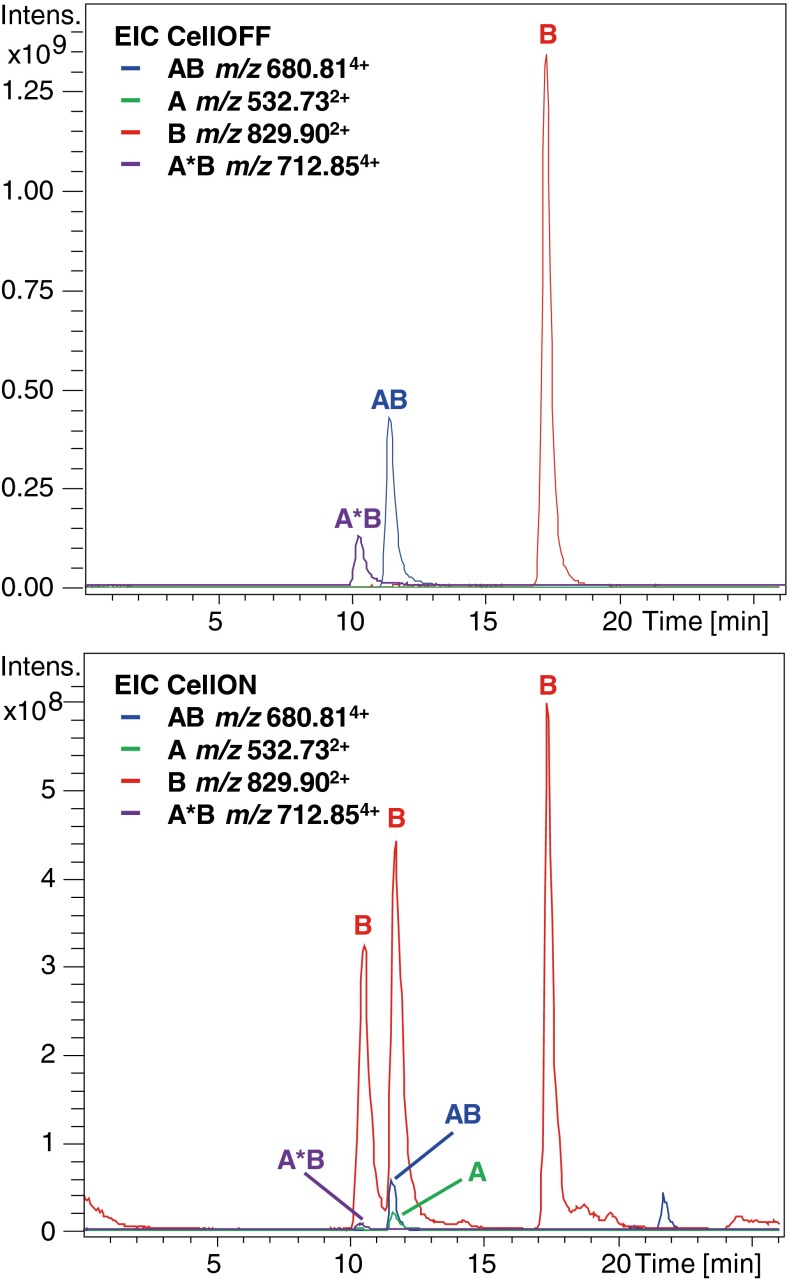
Extracted ion chromatograms (EICs) from the LC-EC-MS analysis of trypsin digested ß-lactoglobulin in CellOFF (top) and CellON (bottom) mode. The disulfide-linked peptide (AB) with disulfide bond C66–C160 was observed at a retention time of 11.5 min with an *m/z* value of 680.81^4+^. The disconnected peptides were detected at the same retention time in CellOFF mode at *m/z* values 532.73^2+^ (A, WENGEC_66_AQK) and 829.90^2+^ (B, LSFNPTQLEEQC_160_HI). A similar disulfide-linked peptide was detected at a retention time of 10.5 min with a missed cleavage in peptide A (i.e., A*B, at an *m/z* value of 712.85^4+^). Hence, peptide B was also detected at a retention time of 10.5 min in CellON mode. Note that peptide B was also detected at a retention time of 17.5 min in both modes, indicating the partial absence of this disulfide bond in ß-lactoglobulin

In order to minimize disulfide reshuffling and maintain the advantages offered by trypsin, proteolysis was performed at pH 5.5. It was found that digestion was still sufficient at this pH, supplemented with acetonitrile for protein unfolding. In the case of ß-lactoglobulin (18.3 kDa, five cysteines and two disulfide bridges), a sequence coverage of 93% was obtained with a low number of missed cleavages (see Supplemental Material, Table S2 and Figure S3). Digestion of ß-lactoglobulin with trypsin under non-reducing conditions was expected to result in the formation of two disulfide-linked peptides, one with an interchain disulfide bond and one with an intrachain disulfide bond. In Table 1, peptide sequences are shown of the expected and detected disulfide-linked and disconnected species in CellOFF and CellON mode, respectively. In CellOFF mode, the disulfide-linked peptide containing the C66–C160 disulfide bond eluted at a retention time of 11.5 min (see Figure 2). Notably, at 17.5 min a peak corresponding to the disconnected form of the peptide LSFNPTQLEEQC_160_HI (B peptide) is also present in CellOFF mode. This illustrates the advantage of the hyphenation of EC-MS with a LC separation: the disconnected or, in this case, reduced form of the peptide is also present at a different retention time than the disulfide-linked peptide, implying that not all ß-lactoglobulin species *originally* contained the C66–C160 disulfide bond. The difference in retention time in CellOFF mode proves that the absence of the disulfide bond is not an artifact from the LC-EC-MS analysis. The additional peak at 21.9 min in the EIC trace of the CellON run was observed with a monoisotopic *m/z* value of 680.04^4+^ and is not related to the disulfide-linked peptide. Moreover, the free cysteine, C121, is not involved in a disulfide bond and most probably cannot form interprotein disulfide bonds because of limited accessibility [28].

**Table 1:** Overview of the Detected Disulfide-Linked Peptides and Disconnected Peptides in CellOFF and CellON Mode, Respectively, from Digests of ß-Lactoglobulin and Ribonuclease B

Protein	DSB#	C#-C#	Disulfide-linked peptide sequence CellOFF	Disulfide-linked peptide CellOFF	Disconnected peptide CellON
ß-lactoglobulin (trypsin)	B1	C66-C160	WENGEC_66_AQK LSFNPTQLEEQC_160_HI	✓	nd ^a^ ✓
	B2	C106-C119	YLLFC_106_MENSAEPEQSLAC_119_QC_121_LVR	✓	✓ ^b^
Ribonuclease B (trypsin)	R1 R4	C26-C84 C65-C72	QHMDSSTSAASSSNYC_26_NQMMK NVAC_65_K NGQTNC_72_YQSYSTMSITDC_84_R	*nd*	nd ^a^ nd nd
	R2 R3	C40-C95 C58-C110	C_40_KPVNTFVHESLADVQAVC_58_SQK YPNC_95_AYK HIIVAC_110_EGNPYVPVHFDASV	✓	✓ nd ^a^ ✓
Ribonuclease B (trypsin/Glu-C)	R1 R4	C26-C84 C65-C72	QHMDSSTSAASSSNYC_26_NQMMK NVAC_65_K NGQTNC_72_YQSYSTMSITDC_84_RE	✓	✓ nd nd ^a^
	R2	C40-C95	C_40_KPVNTFVHE YPNC_95_AYK	✓	✓ ✓
	R3	C58-C110	SLADVQAVC_58_SQK HIIVAC_110_E	✓	✓ nd ^a^

nd = not detected.

^a^ Only MS1 data available due to low intensity.

^b^ Reduced form of peptide with intrachain disulfide bond.

**Figure 3:**
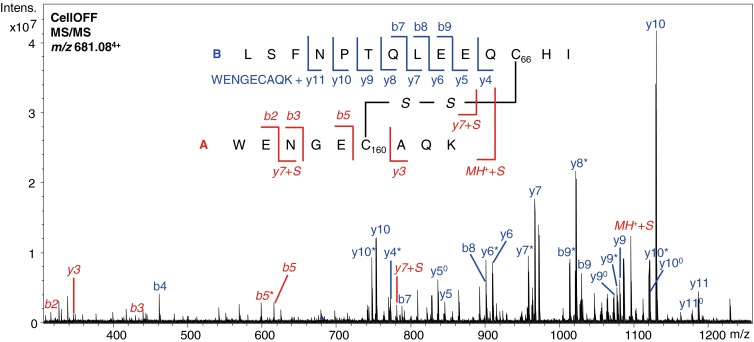
Annotated MS/MS spectrum of the disulfide-linked peptide observed at *m/z* value 681.08^4+^ containing disulfide bond B1 resulting from trypsin digested ß-lactoglobulin. Fragments from peptide A are indicated in red italics, fragments from peptide B are indicated in blue. ^0^Ions resulting from a neutral loss of H_2_O. *Ions resulting from a neutral loss of NH_3_

In the CellON mode, the intensity of the disulfide-linked peptide (AB) decreased significantly and peaks of the disconnected peptides (i.e., A and B) appeared at the same retention time, indicating reduction of the C66–C160 disulfide bond. A peak of disconnected peptide B also appeared at a retention time of 10.5 min. This is probably the result of reduction of the C66–C160 disulfide-linked peptide in which peptide A contains a missed cleavage (WENGEC_66_AQKK); hence, there is no peak appearing of disconnected peptide A at this retention time. The C66–C160 disulfide-linked peptide with the missed cleavage was detected in CellOFF mode (*m/z* value 712.85^4+^), but its intensity was too low to be selected for MS/MS fragmentation. Further confirmation of the presence and location of the C66–C160 disulfide bond was obtained from the MS/MS spectrum of the disulfide-linked peptide (*m/z* value 681.08^4+^), see Figure 3. This spectrum contains a series of y ions resulting from backbone cleavage of peptide B with a mass shift of 1061.42 Da, which corresponds with the mass of peptide A and the presence of a disulfide bond. Furthermore, characteristic ions containing an additional sulfide ( + 32 Da), resulting from S–C cleavage of peptide A, are also present in the spectrum.

The second disulfide bridge of ß-lactoglobulin (C106–C119) is present within a tryptic peptide (i.e., intrachain). Note that this peptide actually contains three cysteines C106, C119, and C121, and based on the information in the uniprot database (www.uniprot.org) the disulfide bridge is present between the C106 and C119 residues. In CellOFF mode, this peptide was detected with the C106–C119 disulfide bond intact (*m/z* value 882.40^3+^), and in CellON mode this disulfide bond was reduced, leading to detection of the reduced peptide (*m/z* value 883.07^3+^). The reduction was confirmed by the shift in the isotope pattern towards a higher *m/z* value. However, the presence of overlapping isotope patterns from the oxidized and reduced species indicates that the reduction was incomplete (see Supplemental Material, Figure S4). In order to confirm the location of the disulfide bond, additional MS/MS data was required.

**Figure 4:**
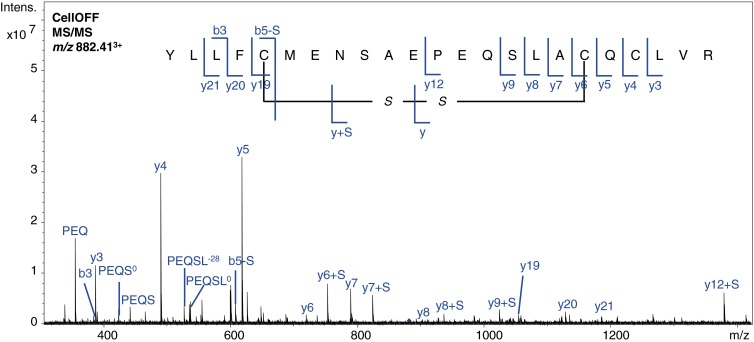
Annotated MS/MS spectrum of the disulfide-linked peptide observed at *m/z* value 882.41^3+^ containing disulfide bond B2 resulting from trypsin digested ß-lactoglobulin. ^0^Ions resulting from a neutral loss of H_2_O

The MS/MS spectrum of the peptide containing the C106–C119 disulfide bond obtained in CellOFF mode (Figure 4) showed backbone cleavage mainly at the peptide termini where the cysteine residues are located. Again, several characteristic ions indicative of the presence of a disulfide bond were present in the spectrum that showed either a loss of a sulfide (–32 Da) or an additional sulfide ( + 32 Da), resulting from C–S cleavage. The fact that the y_6_ (C119), y_7_, y_8_, y_9_, and y_12_ ions all contained an additional sulfide whereas the y_3_, y_4_ (C121), and y_5_ ions did not, points to the disulfide bond being present between C106 and C119 rather than C106 and C121. Some of the y ions (y_6_, y_7_, and y_8_) were also present with a loss of –2 Da, which is a mass shift that has also been observed in other studies and has been contributed to the formation of a double bond during fragmentation [24].

**Figure 5:**
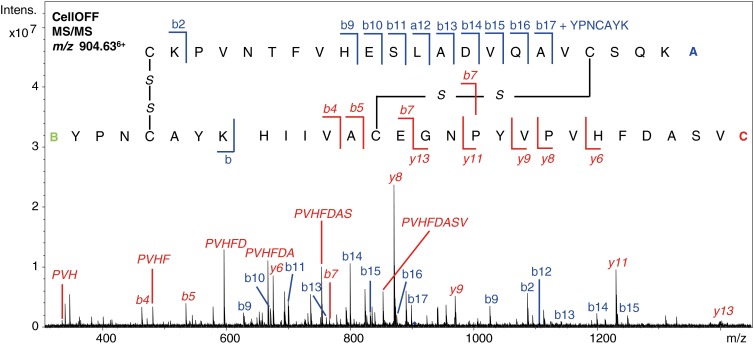
Annotated MS/MS spectrum of the disulfide-linked peptide observed at *m/z* value 904.63^6+^ containing disulfide bond R2 and R3 resulting from trypsin digested ribonuclease B. Fragments from peptide A are indicated in blue and fragments from peptide C are indicated with red italics

As a second example, ribonuclease B (15 kDa, eight cysteines, and four disulfide bridges) was analyzed following the same methodology. In theory, digestion of this protein in its oxidized state using trypsin would result in two disulfide-linked peptides, each consisting of three peptides linked via two disulfide bridges (see Table 1). In practice, a sequence coverage of 40% was obtained and only one of the disulfide-linked peptides was detected (see Supplemental Material, Table S5 and Figure S6). In Figure 5, the MS/MS spectrum is shown of the disulfide-linked peptide consisting of three peptides connected via the C40–C95 and C58–C110 disulfide bonds. From this data, the connectivity of the disulfide bridges can be derived. Backbone cleavage of peptide A resulted in a b-ion series with a mass shift of 855.36 Da that indicates that peptide B is still attached via a disulfide bridge between C40 and C95. Fragments of peptide B itself were not present in the spectrum. Several b- and y-type ions of peptide C were detected, including a C–S cleavage product, which confirms the identity and presence of this peptide in the disulfide-linked peptide and also that it is linked to peptide A with a disulfide bridge between C58 and C110.

**Figure 6:**
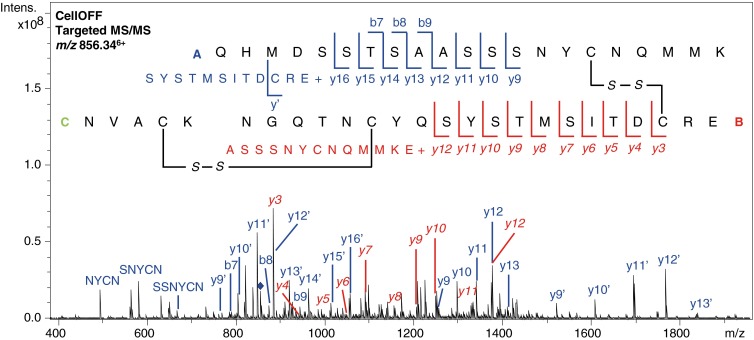
Annotated MS/MS spectrum of the disulfide-linked peptide observed at *m/z* value 856.34^6+^ containing disulfide bond R1 and R4 resulting from trypsin/Glu-C digested ribonuclease B. Fragments from peptide A are indicated in blue and fragments from peptide B are indicated with red italics

Unfortunately, the other disulfide-linked peptide containing the C26–C84 and C65–C72 disulfide bonds was not detected in the trypsin digest and neither were the related disconnected peptides in CellON mode, which is probably due to resistance to proteolytic digestion of ribonuclease B [29]. Therefore, ribonuclease B was subjected to sequential trypsin and Glu-C digestion, to increase protein coverage and the likelihood of detecting the disulfide bridges. Using this alternative approach, the sequence coverage increased to 82% and all four disulfide bonds were detected (see Supplemental Material, Table S7 and Figure S8). Two of the identified disulfide-linked peptides consisted of two peptides connected via one disulfide bond (see Table 1), which are the same disulfide bonds that were also detected in the trypsin digest. The two remaining disulfide bonds were detected in one disulfide-linked peptide consisting of three peptides (see Figure 6 for the annotated MS/MS spectrum). For this disulfide-linked peptide, fragment ions resulting from double C–C cleavage were obtained that indicate the connectivity of the cysteines. Annotated spectra of the other identified disulfide-linked peptides can be found in the Supplemental Material, Figure S9 and Figure S10. The obtained fragmentation patterns of such large, cross-linked peptides are very dense and complex, which shows the value of high resolution mass spectrometry for this application. Often, the isotope patterns of precursor ions or fragment ions, potentially with different charge states, overlap, but these can still be distinguished because of the high resolving power of the FTICR instrument (see Supplemental Material, Figure S11 for several exemplary zoomed-in spectra). This is highly beneficial for the sequence determination and identification of disulfide-linked peptides.

Finally, it was evaluated whether (undesired) side reactions had occurred in the EC cell under the applied conditions, such as possible oxidation or peptide cleavage at tryptophan and tyrosine. This was done by performing database searches that included oxidation of methionine or cysteine as variable modifications on the data obtained from the CellOFF and CellON analyses. In addition, a nonspecific database search was performed to check for nonspecific cleavage products. The search results indeed showed methionine oxidations, however, the same oxidized methionines were detected when the electrochemical cell was switched off. Similar to this, several non-tryptic peptides that had been cleaved at various amino acids were detected in both the CellOFF and CellON analyses, so these are most likely due to nonspecific cleavage of trypsin rather than to electrochemically-induced cleavages. Additionally, no artificial intra- or interprotein disulfide bonds were observed in both proteins.

## Possible Applications

This approach will be applied for disulfide bond characterization of more complex proteins. Digestion of such a disulfide-dense protein results in disulfide-linked peptides that consist of multiple peptides, often more than two, which are linked by multiple disulfide bonds, even if nonspecific proteases are used. Currently available software for disulfide bond identification using other LC-MS-based approaches is not equipped for identification of disulfide-linked peptides consisting of more than two peptides. Here, the advantage of online LC-EC-ESI-MS is clear because the disconnected peptides resulting from the reduction of one disulfide-linked peptide will have the same retention time in the CellON run, which facilitates the identification of this type of complex disulfide-linked peptides.

An example of such a complex protein that contains many disulfide bonds is the NOTCH3 protein. Mutations in the *NOTCH3* gene can cause the genetic disease cerebral autosomal dominant arteriopathy with subcortical infarcts and leukoencephalopathy (CADASIL). *NOTCH3* mutations in CADASIL are typically missense mutations involving a cysteine residue. This leads to a numerical cysteine alteration in one of the 34 consecutive epidermal growth factor-like repeat domains (EGFr) of the NOTCH3 protein [30]. Each EGFr domain typically contains three disulfide bonds that are in very close proximity to each other. In CADASIL, the mutated NOTCH3 EGFr contains either five or seven cysteines instead of the usual six, leading to protein misfolding and toxic accumulation in the (cerebro)vasculature. Disulfide bond formation in this mutated EGFr is believed to be disrupted because of the presence of the unpaired cysteine. The here-proposed technique could lead to important new insights into how cysteine-altering mutations affect disulfide bridges and therefore the pathogenesis of CADASIL.

## Conclusions

The characterization of protein disulfide bonds is challenging in terms of complexity and interpretation of MS and MS/MS data. The application of online LC-EC-ESI-MS offers several advantages over other techniques, most importantly through the additional retention time information obtained from “CellON” and “CellOFF” mode experiments. The use of a LC separation in combination with EC-ESI-MS is advantageous because the link between the disulfide-linked peptide and the disconnected peptides is conserved in the LC retention time dimension, thus facilitating the identification of the actual protein disulfide bonds. The integration of an online LC separation in combination with a bottom-up approach broadens the range of proteins that can be assayed. With the online LC-EC-MS system, different types of disulfide-linked peptides were characterized (i.e., with intrachain and interchain disulfide bonds). All disulfide bonds in ß-lactoglobulin were identified and localized, and it was shown that the disulfide bond between C66 and C160 was only partly present in the protein based on retention time information. In the case of ribonuclease B, which is known to be resistant to proteolytic digestion, a double digestion approach with trypsin and Glu-C was applied to achieve more complete digestion. With this approach, all four disulfide bonds of ribonuclease B were characterized.

## Electronic supplementary material

ESM 1 (DOCX 576 kb)
