# Supplementary material for: In-Depth Characterization of Protein Disulfide Bonds by Online Liquid Chromatography-Electrochemistry-Mass Spectrometry
Source: J Am Soc Mass Spectrom. 2015 Sep 14;27:50–8. doi: 10.1007/s13361-015-1258-z (PMC4686567; doi:10.1007/s13361-015-1258-z)
Supplement: Supplementary file 1 — (DOCX 576 kb) [file 13361_2015_1258_MOESM1_ESM.docx]

# Supporting information


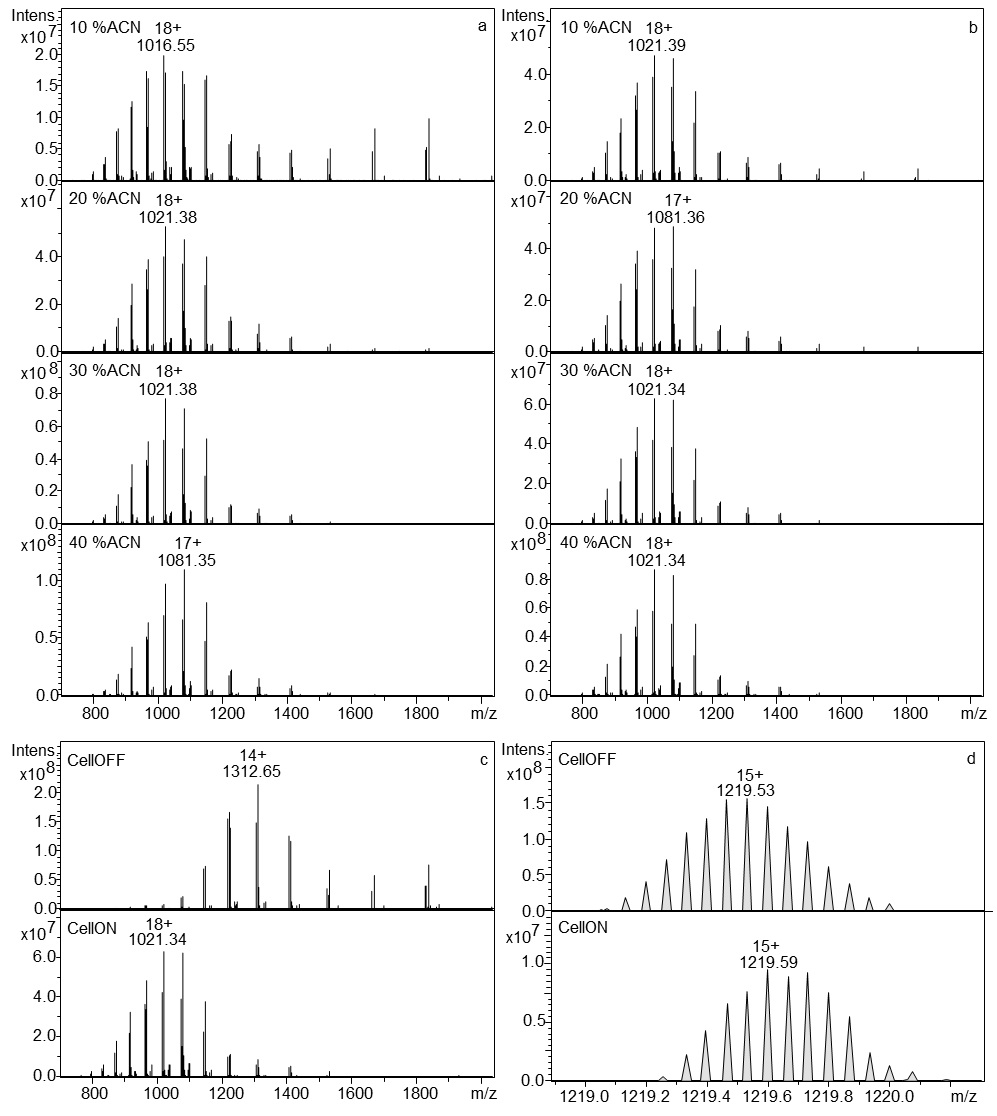


Figure S1. FIA–EC–FTICR MS spectra acquired for intact ß-lactoglobulin under varying electrochemistry conditions. The MS spectra acquired with different percentages of acetonitrile in 0.5 % formic acid in the mobile phase with the cell switched on using the default (a) and optimized (b) electrochemistry conditions shows that with the optimized conditions, protein unfolding is now achieved regardless of the percentage of acetonitrile used. Figure S1c and S1d (zoom) depict spectra obtained in 30 % acetonitrile and 0.5 % formic acid with the CellOFF (top) and CellON (bottom) that clearly show a shift towards higher charge state and a mass shift to higher *m/z* upon reduction of the disulfide bonds.

Table S2. Identified trypsin peptides from ß-lactoglobulin in CellOFF and CellON mode.

| CellOFF | CellON | *m/z* | Missed cleavages | C#–C# | Sequence |
| --- | --- | --- | --- | --- | --- |
| ✓ | ✓ | 467.29 2+ | 0 | - | LIVTQTMK |
| ✓ | ✓ | 337.20 2+ | 0 | - | GLDIQK |
| ✓ | ✓ | 908.49 3+ | 0 | - | VAGTWYSLAM(oxidation)AASDISLLDAQSAPLR |
| ✓ | ✓ | 771.78 3+ | 0 | - | VYVEELKPTPEGDLEILLQK |
| 🗶 | 🗶^a^ | 532.73 2+ | 0 | - | WENGEC_66_AQK |
| ✓ | ✓^b^ | 680.81 4+ | 0 | C66–C160 | WENGEC_66_AQK linked to LSFNPTQLEEQC_160_HI |
| ✓ | ✓ | 337.72 2+ | 0 | - | IPAVFK |
| ✓ | ✓ | 458.75 2+ | 0 | - | IDALNENK |
| ✓ | ✓ | 596.36 2+ | 0 | - | VLVLDTDYK |
| 🗶 | ✓^c^ | 883.10 3+ | 0 | - | YLLFC_106_MENSAEPEQSLAC_119_QC_121_LVR |
| ✓ | ✓^c^ | 882.40 3+ | 0 | C106–C119 | YLLFC_106_MENSAEPEQSLAC_119_QC_121_LVR |
| ✓ | ✓ | 623.32.2+ | 0 | - | TPEVDDEALEK |
| ✓ | ✓ | 419.25 2+ | 0 | - | ALPMHIR |
| ✓ | ✓ | 829.93 2+ | 0 | - | LSFNPTQLEEQC_160_HI |

^a^Only MS data, ^b^Low intensity MS/MS spectrum, ^c^Mixture spectrum in CellON analysis


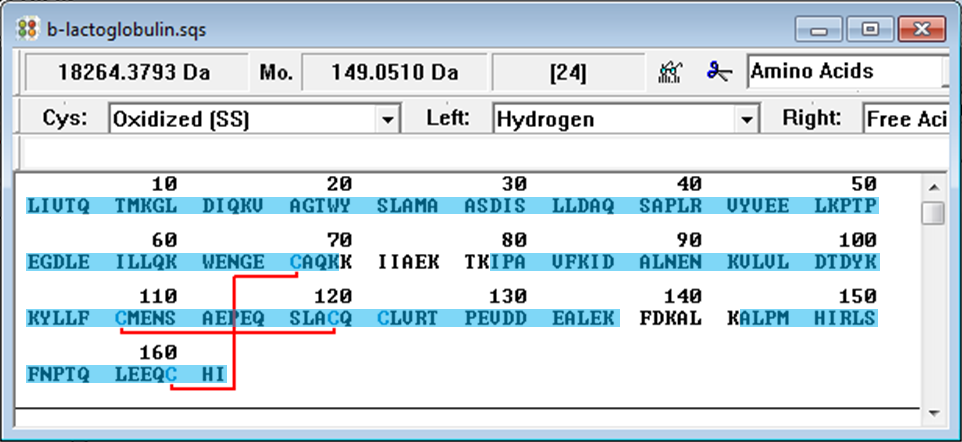


Figure S3. Amino acid sequence of ß-lactoglobulin indicating the connectivity of the disulfide bonds (red lines) and sequence coverage ((93 %, highlighted blue) after trypsin digestion.


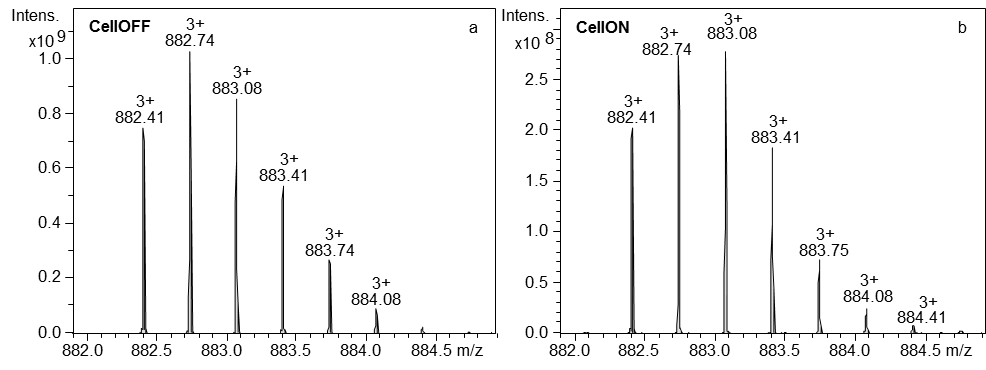


Figure S4. MS spectra obtained in CellOFF (a) and CellON (b) mode of a trypsin peptide from ß-lactoglobulin with an intrachain disulfide bond: YLLFC_106_MENSAEPEQSLAC_119_QC_121_LVR, m/z 883.10 3+ (reduced), m/z 882.40 3+ (oxidized). A shift in the isotope pattern towards higher *m/z* is visible after reduction of the disulfide bond in CellON mode.

Table S5. Identified trypsin peptides from ribonuclease B in CellOFF and CellON mode.

| CellOFF | CellON | *m/z* | Missed cleavages | C#–C# | Sequence |
| --- | --- | --- | --- | --- | --- |
| 🗶 | ✓ | 801.74 3+ | 1 | - | C_40_KPVNTFVHESLADVQAVC_58_SQK |
| 🗶 | 🗶^a^ | 429.70 2+ | 0 | - | YPNC_95_AYK |
| 🗶 | ✓ | 723.03 3+ | 0 | - | HIIVAC_110_EGNPYVPVHFDASV |
| ✓ | 🗶^a^ | 904.61 6+ | 1 | C40–C95 C58–C110 | C_40_KPVNTFVHESLADVQAVC_58_SQK linked to  YPNC_95_AYK and HIIVAC_110_EGNPYVPVHFDASV |

^a^Only MS data


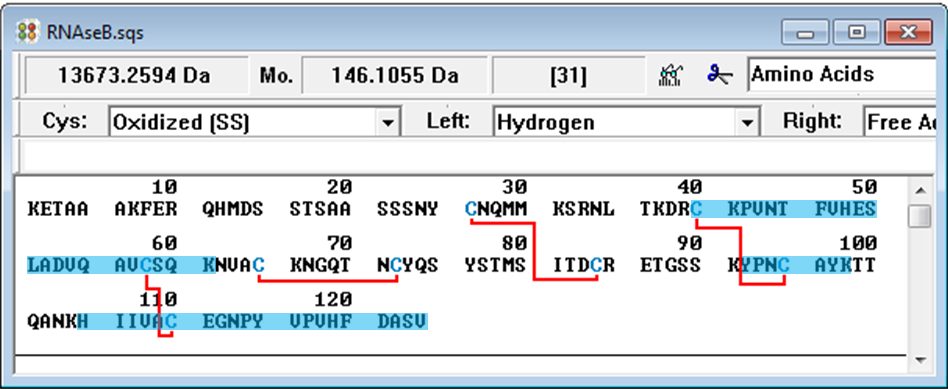


Figure S6. Amino acid sequence of ribonuclease B indicating the connectivity of the disulfide bonds (red lines) and sequence coverage (40 %, highlighted blue) after trypsin digestion.

Table S7. Identified trypsin-GluC peptides from ribonuclease B in CellOFF and CellON mode.

| CellOFF | CellON | *m/z* | Missed cleavages | C#–C# | Sequence |
| --- | --- | --- | --- | --- | --- |
| 🗶 | ✓ | 769.97 3+ | 1 | - | QHMDSSTSAASSSNYC_26_NQMMK |
| 🗶^a^ | ✓ | 587.29 2+ | 1 | - | C_40_KPVNTFVHE |
| 🗶 | ✓ | 624.83 2+ | 1 | - | SLADVQAVC_58_SQK |
| 🗶 | ✓ | 429.70 2+ | 0 | - | YPNC_95_AYK |
| ✓ | ✓ | 572.78 2+ | 0 | - | GNPYVPVHFD |
| ✓ | ✓ | 701.36 2+ | 1 | - | GNPYVPVHFDASV |
| ✓^b,c^ | 🗶^b^ | 857.04 6+ | 3 | C26-C84  C65-C72 | QHMDSSTSAASSSNYC_26_NQMMK and NVAC_65_K  linked to NGQTNC_72_YQSYSTMSITDC_84_RE |
| ✓ | 🗶^a^ | 676.99 3+ | 1 | C40–C95 | C_40_KPVNTFVHE linked to YPNC_95_AYK |
| ✓ | ✓ | 677.35 3+ | 1 | C58–C110 | SLADVQAVC_58_SQK linked to HIIVAC_110_E |

^a^Only MS data, ^b^Low intensity MS/MS spectrum, ^c^Confirmed by targeted MS/MS analysis


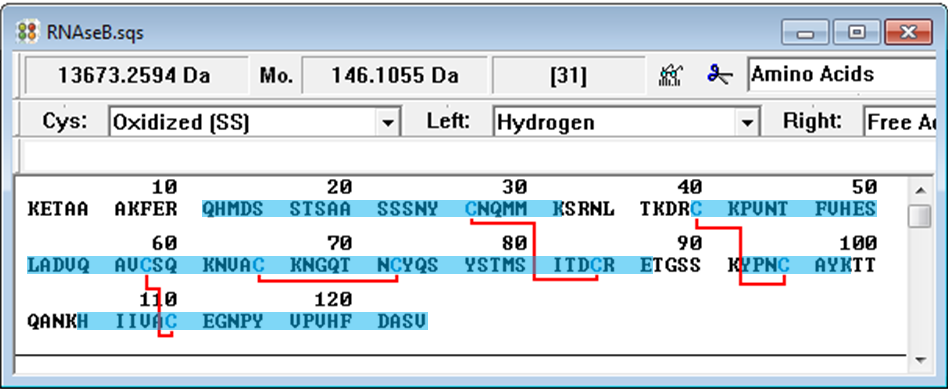


Figure S8. Amino acid sequence of ribonuclease B indicating the connectivity of the disulfide bonds (red lines) and sequence coverage (highlighted blue) after trypsin-GluC digestion.


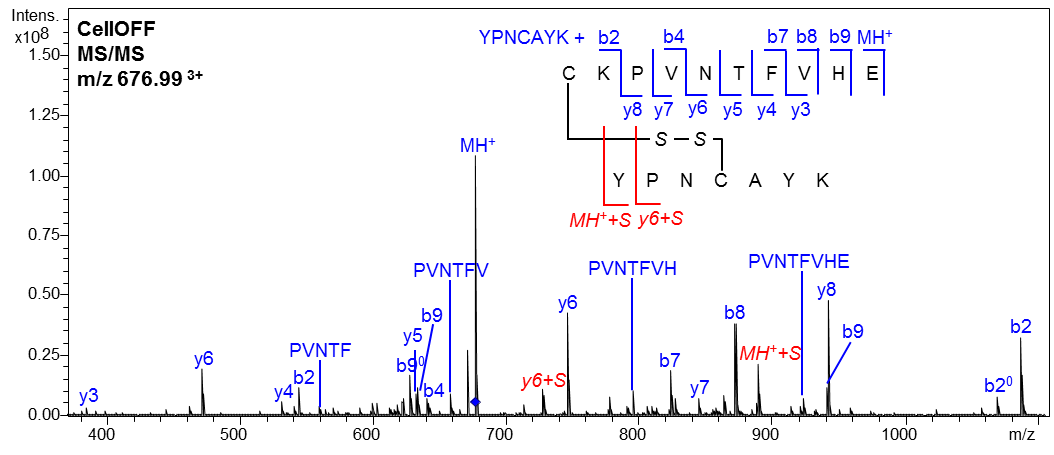
Figure S9. Annotated MS/MS spectrum of the disulfide-linked peptide observed at *m/z* value 676.99 3+ containing disulfide bond R2 resulting from trypsin-GluC digestion of ribonuclease B. Fragment ions indicated with a ^0^ result from a neutral loss of H_2_O.


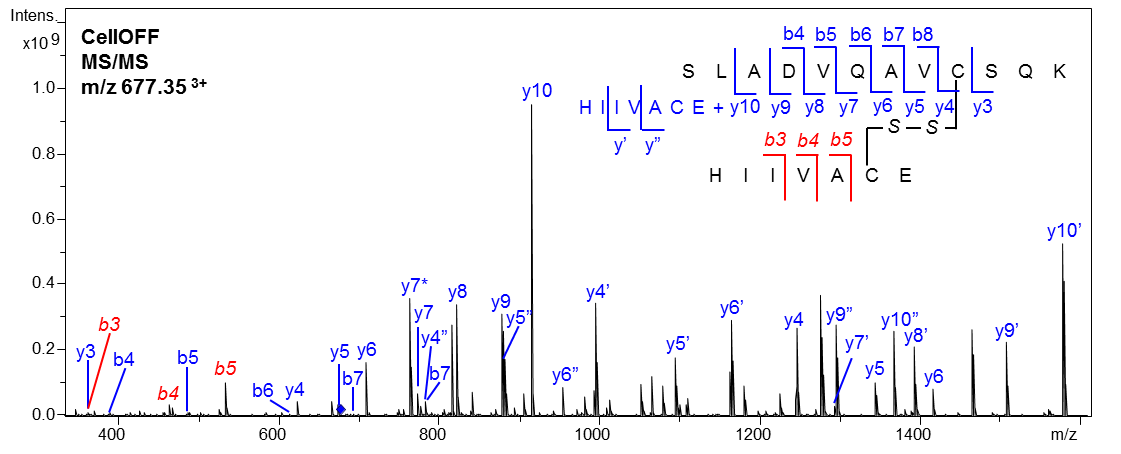
Figure S10. Annotated MS/MS spectrum of the disulfide-linked peptide observed at *m/z* value 677.35 3+ containing disulfide bond R3 resulting from trypsin-GluC digestion of ribonuclease B.


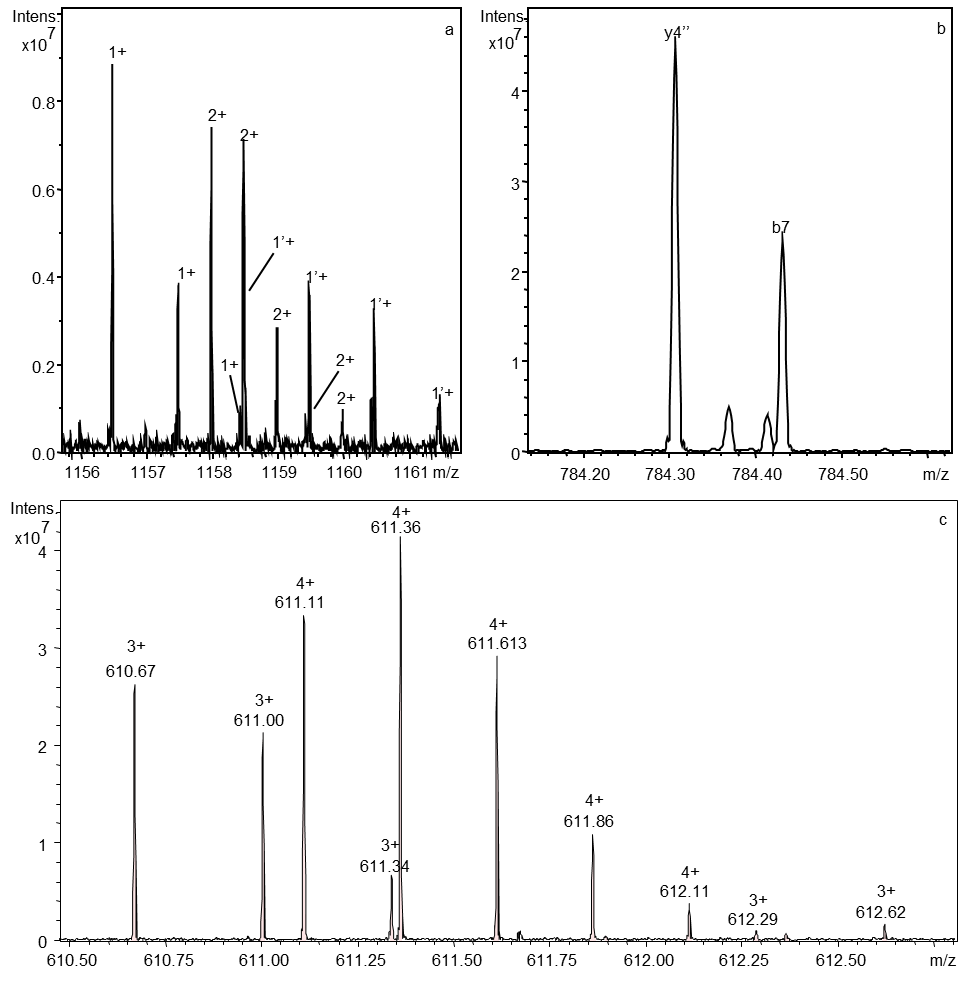


Figure S11. Zoomed-in spectra showing the advantage of high resolution MS measurements. (a) Zoom-in from the MS/MS spectrum of the disulfide-linked peptide observed at *m/z* value 856.34^6+^ containing disulfide bond R1 and R4 resulting from trypsin-gluC digested ribonuclease B. The y8^2+^ isotope pattern overlaps with two singly charged ion species (labeled 1+ and 1’+), but can be clearly distinguished. (b) Zoom-in from the MS/MS spectrum of the disulfide-linked peptide with *m/z* 677.35 3+ containing disulfide bond R3 resulting from trypsin-GluC digestion of ribonuclease B. The y4’’ and b7 fragment ions, with *m/z* values of 784.31 and 784.43, respectively, are well resolved. (c) Zoom-in from a MS spectrum of the CellOFF analysis of trysin digested ß-lactoglobulin. The overlapping isotope patterns of two different ion species with chargestates of 3+ and 4+ can be clearly distinguished.
